# Adeno-Associated Virus Vectors: Principles, Practices, and Prospects in Gene Therapy

**DOI:** 10.3390/v17020239

**Published:** 2025-02-09

**Authors:** Limor Zwi-Dantsis, Saira Mohamed, Giulia Massaro, Emad Moeendarbary

**Affiliations:** 1 Department of Mechanical Engineering, Roberts Building, University College London, London WC1E 6BT, UK; 2 UCL School of Pharmacy, University College London, London WC1N 1AX, UK

**Keywords:** adeno-associated virus (AAV), gene therapy, vector manufacturing, clinical trials

## Abstract

Gene therapy offers promising potential as an efficacious and long-lasting therapeutic option for genetic conditions, by correcting defective mutations using engineered vectors to deliver genetic material to host cells. Among these vectors, adeno-associated viruses (AAVs) stand out for their efficiency, versatility, and safety, making them one of the leading platforms in gene therapy. The enormous potential of AAVs has been demonstrated through their use in over 225 clinical trials and the FDA’s approval of six AAV-based gene therapy products, positioning these vectors at the forefront of the field. This review highlights the evolution and current applications of AAVs in gene therapy, focusing on their clinical successes, ongoing developments, and the manufacturing processes required for the rapid commercial growth anticipated in the AAV therapy market. It also discusses the broader implications of these advancements for future therapeutic strategies targeting more complex and multi-systemic conditions and biological processes such as aging. Finally, we explore some of the major challenges currently confronting the field.

## 1. Introduction

Gene therapy involves introducing genetic material into patients to alter gene or protein expression and is a promising treatment option for a number of diseases such as cancer, inherited disorders, and certain viral infections [1]. Generally, genetic diseases driven by mutations in the human genome are ideal targets for gene therapy. While this technology usually targets disorders with well-defined patho-physiological mechanisms and gene functions, in more recent years gene therapies have been investigated for the treatments of complex multigenic conditions such as Alzheimer’s and Parkinson’s diseases. Gene therapy technology utilizes a vector and its payload to correct genetic diseases in three ways: (1) replacing the defective gene with a functional copy, (2) silencing the mutated version of the gene, or (3) adding or overexpressing a distinct therapeutic gene or synthetic molecule [2]. There are two main gene therapy strategies: ex vivo and in vivo. Ex vivo gene therapy involves the removal of somatic cells or tissues from the patient’s body; specific genes in these cells are genetically modified outside the body and then reintroduced back into the patient [3]. In vivo gene therapy, on the other hand, delivers therapeutic genetic material directly into the somatic cells of the target organs or tissues of the patient’s body, achieving long-lasting expression within the host cells [4,5].

Numerous gene delivery systems, including viral vectors (such as adenoviruses, AAVs, and lentiviruses) [6] and non-viral vectors (such as lipid nanoparticles, cationic polymers, inorganic particles, and conjugate complexes) [1], have been introduced for the gene therapy of human genetic diseases. Non-viral vectors are praised for their low immunogenicity, cost-effectiveness, and flexibility in handling large DNA sizes and targeting specific cells [7] and have shown promising gene transfer results in animals [8]. Nevertheless, non-viral gene delivery systems face challenges such as low transfection efficiency and the limited viability of the transferred cells. Additionally, these systems often lack effective targeting capabilities, which can limit their practical application in therapeutic settings [9]. In contrast, viral vectors provide a robust alternative. Utilizing the virus’s innate capability to penetrate cell nuclei and deliver genetic material, viral vector platforms offer several advantages, including high transfection efficiency, improved safety, and extensive development over decades, which have led to successful clinical outcomes in treating a variety of diseases [10].

Among virus-derived vectors, AAVs have become a prominent vector for in vivo gene transfer and have emerged as one of the most efficient and relatively safe vehicles. AAVs’ low pathogenicity and immunogenicity and wide cellular tropism, combined with their ability to provide sustained transgene expression, make them a preferred vector in gene therapy [11]. Notably, AAVs do not alter the sequence and structure of the genome, as the majority of the genomic material remains episomal, thus reducing the risk of insertion mutagenesis and preventing the introduction of genome instability factors and enhancing safety. However, it is estimated that approximately 0.1% of the wild-type genome integrates with the AAVS1 site in chromosome 19 [12]. Nevertheless, rAAV-mediated gene replacement has been widely used in clinical trials involving a variety of gene therapies to treat various inherited and acquired human diseases, including congenital blindness, hemophilia, neuromuscular disease, and immune disorders, among many others. This successful strategy is now undergoing accelerated clinical and commercial development, with several products recently receiving approval in the USA and Europe [13]. The global market value of AAV therapies reached USD 1.5 billion in 2023 and is expected to peak at USD 22.3 billion in 2029, based on GlobalData forecasts.

This review discusses the foundations and recent breakthroughs in the field of using AAVs for gene therapy, providing a summary of AAV biology, manufacturing, current clinical trials, and challenges. In addition, we review representative target genes and strategies for employing the AAV gene therapy platform for treating aging or age-related diseases.

## 2. Main Review

### 2.1. AAV Structure and Function

The wild-type AAV is a small, non-enveloped virus belonging to the *Parvoviridiae* family, first identified in 1965 as a contaminant of adenovirus preparations. AAV particles are approximately 25 nm in diameter, with a single-stranded DNA (ssDNA) genome of approximately 4.7 kb in size [4,14]. From a structural proteins perspective, the icosahedral capsid is composed of 60 subunits with a mixture of three capsid (Cap) proteins—VP1, VP2, and VP3—at a molar ratio of 1:1:10 which are involved in structural stability, receptor binding, cellular entry, and DNA encapsidation. Additionally, it includes four non-structural replication (Rep) proteins essential for AAV replication, transcriptional regulation, and genome packaging—Rep78, Rep68, Rep52, and Rep40—and the assembly-activating protein (AAP), which provides a scaffold for capsid assembly [15,16]. In addition, a novel frameshift open reading frame in the VP1 region producing the membrane-associated accessory protein MAAP [17] has been identified to play a role in the release of AAV particles from the cell [18].

**Figure 1:**
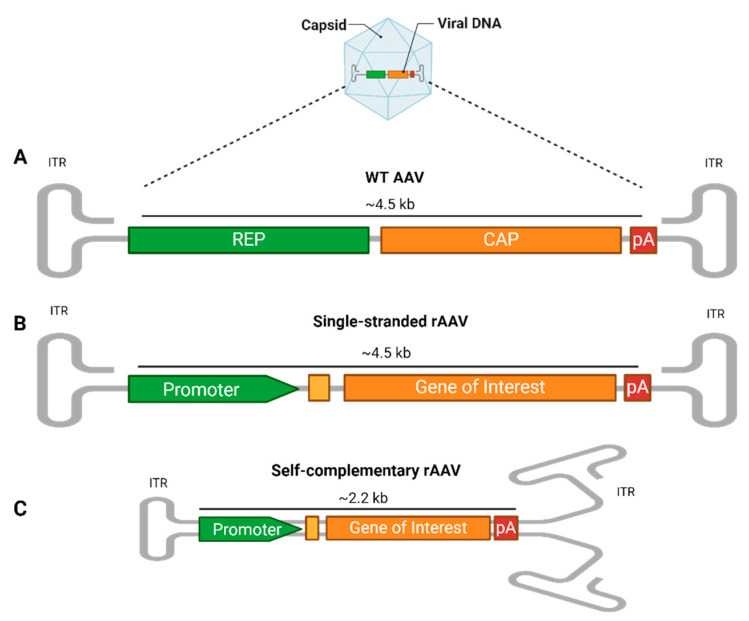
AAV vector structure. (**A**) The wild-type (WT) AAV genome contains the *rep* (replication) and *cap* (capsid) genes, along with regulatory elements like polyadenylation (PolyA). These are flanked by two inverted terminal repeats (ITRs), which form T-shaped hairpin structures critical for genome replication and packaging. (**B**) In single-stranded recombinant AAVs (rAAV), the *rep* and *cap* genes are removed and replaced with a transgene expression cassette that includes a promoter, additional enhancers or a Kozak consensus sequence (yellow box), gene of interest, and PolyA, flanked by ITRs. The foreign DNA packaging capacity is approximately 4.5 kb. (**C**) In self-complementary rAAVs (scAAVs), the 5′ ITR is mutated, allowing the single-stranded genome to fold into a double-stranded structure, reducing the genome size to 2.2 kb but increasing the transduction efficiency. rAAVs can be tailored at the capsid and promoter levels, where the capsid’s serotype determines the tissue tropism, while the promoter drives tissue-specific or widespread transgene expression.

The AAV genome includes the *rep* and *cap* genes, essential for the AAV’s life cycle, flanked by two AAV-specific palindromic 145 bp inverted terminal repeats (ITRs), characterized by T-shaped hairpin structures (Figure 1A). The ITRs play essential roles in host genome integration, vector production, and persistent cell transduction [19,20]. Despite their widespread presence, with over 100 identified natural variants and several serologically distinct serotypes [21], AAV infections are typically harmless and asymptomatic, occurring primarily in early childhood. However, AAVs rely on co-infection with helper viruses like Adenovirus or Herpes simplex virus for replication due to their inherent replication deficiency [4].

For gene therapy applications, recombinant AAVs (rAAVs) are engineered to provide long-term gene expression with limited chromosomal integration [22], thus preserving the host’s genome integrity [6,23]. In the rAAV system, most of the viral genome, including the *rep* and *cap* genes, is replaced by an expression cassette. This cassette contains a gene of interest (GOI), flanked by two ITRs, and features a promoter upstream to initiate expression and a polyadenylation (polyA) tail downstream to enhance the mRNA stability and promote efficient translation. These elements can drive and regulate the expression of the gene of interest in specific cell types. The foreign DNA packaging capacity is approximately 4.5 kb (Figure 1B). This configuration prevents rAAVs from replicating on their own, necessitating external sources of *cap* and *rep* genes to assemble functional virus particles [24]. Consequently, rAAVs are extremely safe vectors for delivering and maintaining transgene expression following a single infection, making them ideal for clinical applications [16].

An innovative modification to the initial recombinant AAV (rAAV) design involves creating a self-complementary rAAV cassette (scAAV). The synthesis of the complementary DNA strand following the translocation of the genome into the nucleus is considered a rate-limiting factor for the transduction efficiency. Therefore, a second generation of vectors with double-stranded DNA have been developed [25]. In this adaptation, the 5′ ITR is altered by deleting the AAV terminal resolution site and D sequence, prompting the single-stranded genome to fold back on itself and form a double-stranded, or self-complementary, structure [26]. This alteration effectively halves the cargo DNA packaging capacity to approximately 2.2 kb (Figure 1C). Despite the reduction in the DNA capacity, this structural transformation significantly enhances the transduction efficiency, potentially increasing it by tenfold or more compared to that of a traditional single-stranded AAV. This increase is due to the elimination of the need for the host cell to convert the single-stranded DNA into double-stranded DNA. Additionally, this change leads to more stable transgene expression, leveraging the intrinsic advantages of the double-stranded format while maintaining similar elements to those in the single-stranded rAAV cassette [27,28].

**Figure 2:**
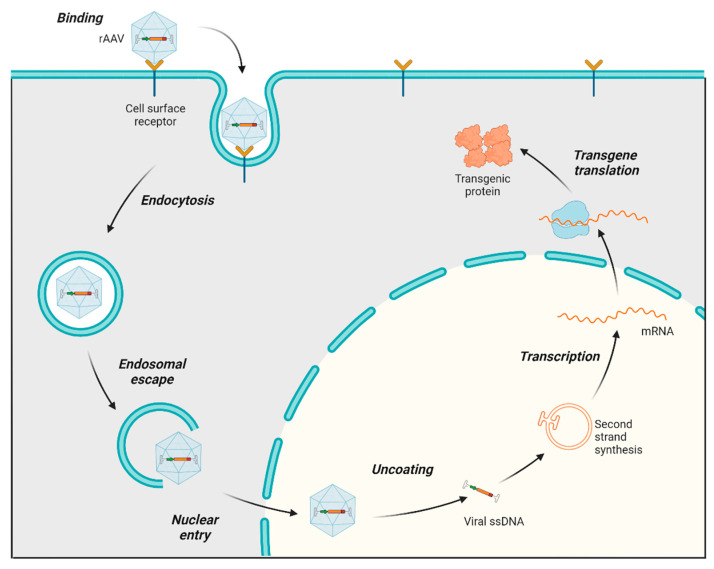
AAV transduction pathway. Main steps in the AAV transduction pathway: The rAAV capsid binds to cell surface receptors, triggering the internalization of the virus via endocytosis, which forms endosomes. Following endosomal escape, the AAV is transported into the nucleus and uncoats. The single-stranded AAV genome converts to double-stranded DNA (except in scAAV, which skips this step for faster transduction). The AAV DNA is then transcribed into mRNA, which exits the nucleus and is translated into therapeutic proteins.

The process of infecting and transducing cells using AAVs involves a sequence of events, starting with the AAV capsid binding to proteoglycan receptors on the cell surface (both primary receptors and coreceptors) of the host. Following this initial interaction, the AAV is internalized into the cell through endocytosis. After undergoing a structural transformation, AAV virions escape the endosome and accumulate in the perinuclear region of the cell [29,30]. Subsequently, they enter the nucleus through the nuclear pore. Once in the nucleus, the virus uncoats, releasing ssDNA, which is converted to double-stranded DNA (dsDNA), followed by transcription, mRNA translation, and the expression of the therapeutic transgene (Figure 2) [31]. AAV genomes mostly exist as circular episomes, although a low frequency of integration into the host chromosomes has been reported [22]. Modifications in the AAV vector design to target specific steps in this transduction pathway can significantly influence the efficiency of transduction. For instance, the phosphorylation of threonine (T) residues exposed on the AAV capsid can impede nuclear transport. Consequently, substituting a threonine residue with valine (T491V or T492V) in serotypes such as AAV2 and AAV6 has been found to enhance nuclear entry and improve the overall transduction efficiency [32].

The most used methods for AAV delivery into various tissues include intravenous (IV), intrathecal (IT), intramuscular (IM), intraocular, and intraperitoneal (IP) injections. IV methods offer systemic distribution, IT methods target the central nervous system, IM methods are used for muscle conditions, intraocular methods are for retinal diseases, and IP methods are employed in animal models for broader exposure. Each method has its advantages and limitations, as described in Table 1.

### 2.2. AAV Serotypes and Tropisms

To date, at least 13 natural serotypes and over 100 variants have been identified and characterized, which allow for the targeting of a broad range of cells and tissues [33]. Depending on their serotype, AAVs can have a specific tropism for certain organs and tissues of the body, with distinct tissue-specific transduction efficiencies [34]. The diversity of AAV serotypes is leveraged in gene therapy to target different types of cells or tissues.

AAV1, initially identified as a contaminant in adenovirus preparations, was the first viral vector to be approved for use in gene therapy [35]. It is distinguished by its inability to bind heparin and its lack of post-translational modifications. Nevertheless, this serotype excels in gene therapy applications due to its sialic acid-based cell entry mechanism and broad efficacy in transducing skeletal muscles, neurons, and various other tissues (such as the heart [36,37], endothelial and vascular smooth muscles [38], and the retina [39]) across multiple species. AAV2, discovered in 1965 and recognized for its affinity for heparan sulfate, is the most studied among all AAVs and utilized in research and clinical trials [40], showing a broad tissue tropism, including for renal tissue [41,42], hepatocytes [43,44], the retina [45,46], the central nervous system (CNS) [47,48], and skeletal muscles [40]. AAV3, initially overlooked as a potential AAV vector for gene therapy due to its low in vitro transduction efficiency in murine cell lines, later revealed significant potential for targeting human liver cancer cells and hepatocytes [49,50,51]. AAV4 and AAV5, with distinct antigenic profiles and unique receptor usages, demonstrate specialized tissue transduction capabilities, such as for the CNS [52] and retinal cells [53]. AAV6 shows high transduction efficiency across various tissues, including airway epithelia [54,55,56] and skeletal muscles [57]. AAV7, AAV8, and AAV9, isolated from non-human primates and humans, exhibit strong tropisms for liver cells and skeletal and cardiac muscles, with AAV9 additionally crossing the blood–brain barrier (BBB) and being the most favored for neurological applications [58]. The newly identified AAV10 and AAV11, displaying broad tissue tropisms and distinct cellular targets, along with AAV12 and AAV13, expanding the understanding of AAVs’ cell entry mechanisms and potential therapeutic applications, showcase the evolving landscape of AAV research [59]. Table 2 provides a comparative overview of AAV serotypes and their tissue specificity.

The selection of an AAV serotype for gene therapy is influenced by specific requirements, including the target tissue, the desired level of gene expression, and the duration of expression [13]. In recent years, innovative engineering strategies such as the creation of mosaic, chimeric, and combinatorial vector libraries have been utilized to develop novel hybrid vectors and serotypes with the aim of enhancing the targeting specificity, efficiency, and safety in gene therapy applications [30]. Techniques like cross-packaging, where the viral genome of one serotype is packaged into the capsid of another type, represent another strategy to increase the transduction efficiency and minimize immune recognition, thus expanding AAVs’ therapeutic potential [60,61]. More recent advancements involve the rational design of novel vectors, where site-specific modifications to the capsid improve the tissue specificity or modify antigenic sites [62,63,64], and direct evolution, where capsid libraries are generated via an iterative selection process of selective pressure and the isolation of variants with specific desired traits [65,66]. This was the case for the neurotropic PHP AAV capsid family (PHP.B and PHP.eB), developed by Deverman and which evolved from the naturally occurring AAV9 serotype, with improved brain penetrance and CNS transduction abilities [67].

### 2.3. AAV Manufacturing

**Figure 3:**
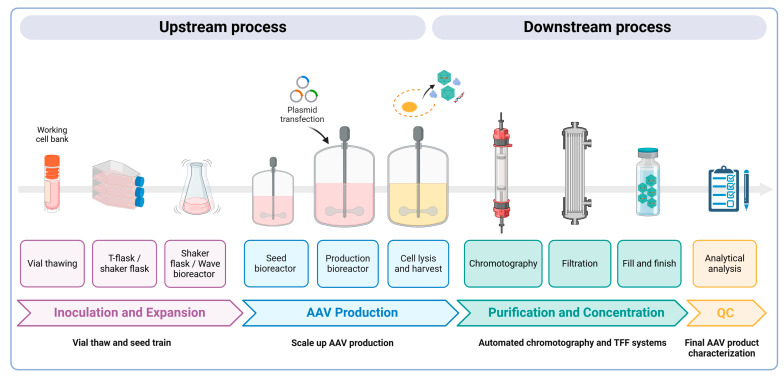
A schematic overview of AAV vector manufacturing. Upstream process: Cells are expanded and transferred into a production bioreactor (50–200 L) based on the scale of AAVs required. A triple transfection process with three plasmids (transgene, packaging, and helper) is then performed to produce AAV vectors designed to deliver therapeutic genes. A few days later, the cells are lysed to release both full and empty AAV vectors, as well as other cellular components. Downstream process: Purification methods are employed, including affinity chromatography and ion exchange chromatography, which are crucial for separating full AAV capsids from empty ones, ensuring high purity and potency. Tangential flow filtration (TFF) is often used for concentration and buffer exchange, ensuring sterility. The final gene therapy product is packaged into vials and undergoes detailed characterization, including assessments of the viral genome titer, purity, and stability to ensure safe, clinical-grade AAV vectors.

The large-scale production of AAV vectors is essential for both preclinical and clinical uses, requiring substantial amounts of highly pure rAAV particles at high yields. As more gene therapies gain approval, the demand for commercial-scale production increases; yet, scaling up these processes remains a significant challenge. Larger doses are necessary to achieve the desired therapeutic effects, which in turn increases manufacturing costs. Strategies must also be employed to improve the production yield and efficacy while minimizing the presence of empty capsids. Empty AAV capsids form due to imbalances during the vector production process. Common reasons include the insufficient availability of the viral genome template, incomplete packaging, or the overproduction of capsid proteins relative to the DNA template. Notably, in the transient transfection of HEK293 cells, empty AAV capsids can account for 50–90% of the total AAV particles generated in a cell culture [68]. These empty capsids are generally considered impurities and can elicit immune responses due to an increased viral load during dosing, thereby reducing the efficacy of gene therapy treatments [69]. Here, we present an overview of the main methodologies for AAV production, purification, and characterization to meet these demands (Figure 3).

#### 2.3.1. Upstream Process

The plasmid-based transient transfection of adherent HEK293 cells (a human embryonic kidney cell line) grown in serum-containing media remains the most commonly used method to produce AAV vectors for preclinical research. This method involves transfecting three plasmids using co-precipitation strategies, such as using calcium phosphate, polyethylenimine (PEI), or cationic lipids [70]. However, this conventional method faces scalability issues, as it would require exponentially increasing the number of culture flasks or cell factories. This makes it impractical due to space, cost, and labor constraints, and it is not compliant with current GMP (Good Manufacturing Practice) standards [71]. To address these challenges, newer methods have been developed that utilize suspension HEK293 cells, which can be grown in animal component-free and antibiotic-free media in various bioreactor types, such as shaker flasks, orbitally shaken bioreactors, wave bioreactors, and stirred tank bioreactors [72,73,74,75,76]. These methods have been shown to produce a high purity and yield of AAV particles, typically exceeding 10^5^ vector genomes per cell and 10^14^ vector genomes per liter of culture [75]. Additionally, the serum-free conditions increase safety by eliminating a potential source of adventitious agents, simplify downstream purification, and reduce analytical testing requirements.

Stable cell lines derived from HeLa cells, such as packaging and producer lines, represent another approach for producing high yields of rAAV particles, comparable to those from transfection methods, while limiting the risk of plasmid DNA contamination and reducing the costs of the GMP-grade starting material [77,78,79]. Packaging cell lines produce rAAVs following either transfection with an AAV construct and co-infection with an Ad helper virus or infection with a recombinant Ad/AAV hybrid helper virus. Producer cells, which contain both AAV *rep*/*cap* genes and the rAAV genome, require just a single infection step with an Ad helper virus for production [80,81]. However, their use in clinical settings is limited due to the complexity of creating and maintaining these lines, including ensuring genetic stability and removing contaminants like wild-type Ad5 from the final product [80,81].

Ongoing efforts are focused on optimizing the upstream processes of AAV production to improve the yield and reduce the presence of empty AAVs. For example, adjusting the ratio of plasmids used in triple transfection protocols, particularly between the packaging plasmid and the plasmid encoding the viral genome, ensures the efficient encapsulation of genetic material while also reducing backbone plasmid contamination [82]. Fine-tuning process parameters, such as the pH, temperature, and ionic strength during production, can further enhance the assembly efficiency. Additionally, efforts have been made to identify small-molecule chemical additives that boost rAAV production or to create a hybrid rep gene, resulting in a 2–4-fold increase in full capsids [82].

#### 2.3.2. Downstream Process

The overall success of AAV clinical manufacturing critically relies on downstream purification steps to generate a final clinical product of a high yield, high potency, and high purity. The purification of rAAVs involves addressing several challenges, including separation from cellular and viral impurities, the removal of empty AAV capsids, and maintaining the potency and stability of the viral particles. Different methods are tailored to these needs, specific to the capsid serotypes and production scales.

A widely used method for rAAV purification in laboratory settings is standard affinity chromatography or density gradient ultracentrifugation using iodixanol or cesium chloride (CsCl). This technique is particularly effective at separating full from empty AAV particles based on their shape, size, and isopycnic point. While the initial round significantly reduces impurities, achieving higher purity may require two or three rounds. Therefore, these methods are time-consuming and not suitable for large-scale production due to challenges in implementing them in a GMP-compliant manner [83,84].

To overcome this limitation, other methods such as affinity or ion exchange chromatography (IEC) are widely used, due to their efficiency, versatility, and scalability. Affinity chromatography relies on specific substrate attachment, mimicking cellular receptors, while IEC utilizes electrostatic interactions between charged AAV capsids and ionized groups in the column matrix. Both methods involve elution by increasing the salt concentration. Chromatography systems offer several advantages, including achieving over 98% purity with a stable yield and purity between batches, automated processing, regenerable resins, dynamic interactions in mild conditions, and an adjustable scale [71,85,86,87]. Notably, while affinity chromatography cannot differentiate between empty and full capsids, IEC effectively removes empty capsids based on their charges, making it crucial for reducing the potential immunogenicity [88,89]. Challenges include suitability issues for certain serotypes, limitations in the resin lifespan, waste generation during regeneration, and potential pollution from improper storage conditions. For large-scale downstream processing, rAAVs need to be recovered from dozens of liters of the cell lysate or medium. Obtaining sufficiently pure rAAV vectors that meet clinical requirements often involves a two-step chromatography process. In the first step, capture chromatography captures rAAVs from raw materials, while the second step, separation chromatography, isolates rAAVs from the elution fraction, primarily to enhance the vector purity by eliminating the remaining contaminants. In some cases, a third or fourth chromatography step is required. Ultracentrifugation could be integrated as a final step to polish the product and eliminate any remaining empty particles after a standard chromatography process.

The purification processes often conclude with a concentration and/or buffer exchange step for the final formulation. Tangential flow filtration (TFF), based on size exclusion, is the predominant technique for this step. TFF is utilized for clarifying whole cell lysates and significantly reducing production volumes, improving virus titration, and saving time [90]. A comparison of AAV purification techniques is shown in Table 3.

Overall, the purification process should achieve high-purity vector preparations for all AAV serotypes, with greater than 90% full particles. Of note, there is great variability in the yield between different serotypes and expression cassettes both at preclinical and GMP grades. Therefore, initial small feasibility batches should be manufactured to predict the yield of the final preparation, contributing to increased production costs. In parallel, emerging technologies, such as machine learning algorithms for process optimization and real-time monitoring systems, are driving further improvements in the precision and efficiency of viral vector manufacturing, reducing the prevalence of empty capsids.

#### 2.3.3. Analytical Analysis

Characterizing the final AAV product involves assessing the viral genome titer, infectious titer (potency), purity (empty capsids, residual host cell and plasmid DNA contaminants, protein contaminants), stability (aggregation), and the identities of the AAV capsid, genome, and therapeutic gene (expression/activity). Over the past decade and a half, mass spectrometry (MS) has become indispensable for analyzing the AAV capsid integrity and post-translational modifications, as well as identifying impurities in vector preparations [91]. However, the application of MS is limited by the need for large substrate volumes and specialized equipment. Differential scanning fluorimetry (DSF or AAV-ID), introduced in 2013 [91] and validated in subsequent studies [92,93], offers cost-effective and high-throughput capabilities suitable for monitoring purification processes. It measures the AAV capsid thermostability (melting temperature, Tm) using SYPRO Orange dye and has been particularly useful in mixed samples and for ensuring batch consistency [94].

While advanced MS and DSF techniques provide the precise characterization and quality control of AAV capsids, addressing contamination in AAV samples such as endotoxins and cellular proteins remains critical for producing safe and effective AAV vectors for clinical use. Endotoxins, particularly lipopolysaccharides from Gram-negative bacteria, are prevalent contaminants in AAV stocks and can induce severe immune reactions in recipients. A new protocol has effectively reduced the endotoxin levels in AAV preparations to below detectable thresholds, without affecting the vectors’ functionality or capsid integrity [95]. The presence of cellular proteins in AAV preparations also poses a significant challenge. Proteomic studies have identified numerous cellular proteins that co-purify with AAV capsids, which may influence the biology and efficacy of AAV vectors. However, not all cellular impurities are harmful; some may even enhance the vector transduction [96]. Thus, purification strategies should consider the potential functional benefits of certain co-purifying proteins. Additionally, other testing for the safety attributes of AAV products, such as the sterility, mycoplasma, and adventitious agents (replication-competent AAVs), must be qualified and validated. An overall testing plan should be in place to ensure that the product meets acceptable limits for identity, strength/potency, quality, and purity.

### 2.4. Clinical Landscape of AAV Gene Therapy

The field of AAV-based gene therapy is evolving rapidly, with more than 300 clinical trials—both completed and ongoing—that involve using AAV drug products to treat 55 diseases conducted to date [21,97]. This extensive research has led to multiple approvals across the United States (US) and Europe. The first phase I clinical trial using an AAV product was conducted in the early 2000s, where the safety and efficacy of an AAV2 vector were assessed in cystic fibrosis patients [98,99,100]. While the trials failed to meet their therapeutic endpoints and did not demonstrate a significant increase in the CFTR expression levels, they provided valuable insights into the challenges of an AAV-based therapy, such as limited efficacy due to the insufficient transduction of the target cells and hindered transgene expression due to an immune response. Nevertheless, the trials confirmed the therapy was safe, paving the way to the future use of AAV vectors as a safer and more flexible alternative to adenovirus-based therapies. The lesson learnt from the cystic fibrosis trials led the way to the approval in Europe of the first AAV gene therapy product, Glybera, for patients with lipoprotein lipase deficiency [101,102,103]. A single-dose administration of Glybera resulted in the sustained expression of the therapeutic protein and a long-term reduction in the occurrence and severity of acute pancreatitis and hospitalization episodes. However, uniQure did not apply for a renewal of its marketing authorization, and it was discontinued five years later due to its high cost, despite its good safety profile [104]. In 2017, Luxturna (voretigene neparvovec-rzyl) became the first AAV-based ocular gene therapy approved by the US FDA, designed to improve functional vision in patients with an inherited retinal disease caused by mutations in the RPE65 gene, affecting both adults and children with vision loss [105]. In May 2019, the FDA approved Zolgensma (onasemnogene abeparvovec) an AAV-based gene therapy drug for the treatment of spinal muscular atrophy [106]. In 2022, the European Medicines Agency (EMA) approved Upstaza (eladocagene exuparvovec), an AAV2-based gene therapy to treat aromatic L-amino acid decarboxylase (AADC) deficiency. In the same year, the FDA approved Hemgenix (etranacogene dezaparvovec-drlb) for the treatment of adults with hemophilia B (congenital factor IX deficiency). Notably, in 2023, seven gene therapies were approved by the FDA, including two AAV products: Sarepta Therapeutics’ Elevidys (delandistrogene moxeparvovec-rokl) for treating ambulatory pediatric patients with Duchenne muscular dystrophy (DMD) and BioMarin’s Roctavian (valoctocogene roxaparvovec) for treating hemophilia A (factor VIII deficiency). In April 2024, the FDA approved Pfizer’s BEQVEZ™ (fidanacogene elaparvovec-dzkt) as a one-time gene therapy for adults with hemophilia B. Table 4 summarizes the AAV gene therapies approved by the FDA. These approvals represent significant advances in the field of gene therapy.

The majority of clinical trials fall into five broad therapeutic areas, where promising results have been observed in patients with eye disorders, blood disorders, neurological disorders, lysosomal storage disorders, and muscular diseases. However, the market is expected to grow in the near future as new approaches and delivery methods continue to emerge. Here, we discuss recent advancements and how they have the potential to improve treatments and outcomes for patients with various diseases.

#### 2.4.1. Eye Diseases/Disorders

Inherited eye disorders often lead to retinal degeneration, progressively harming photoreceptor cells and causing vision loss [107]. A significant breakthrough in treating these conditions was the development of Luxturna for Leber congenital amaurosis caused by the RPE65 mutation, paving the way for clinical trials on various untreatable hereditary eye diseases. The long-term expression of Luxturna was evaluated in a 4-year follow-up study in phase III patients, with positive efficacy and safety results suggesting a long-term benefit. Currently, gene therapy trials focusing on the retina represent the largest segment (34%) in clinical research, with 30 trials using AAV vectors for targeted delivery. Notably, 20% of these trials are in the advanced stages (phase III or II/III), the highest proportion across all disease groups studied [97]. While most trials have utilized AAV serotypes 2, 4, 5, and 8, variants like AAV.7m8 and AAV2tYF have also been explored, with no severe AAV-related complications reported [108,109]. Some patients have shown improvements, indicating the potential for therapeutic benefits. However, the outcomes vary, and the durability of these gene therapies remains uncertain. Since 2017, when Luxturna was approved, no new retinal therapy has been approved despite the numerous clinical trials. For some, the initial therapeutic success decreased over time [108,110,111], possibly due to continued retinal deterioration or immune system reactions [112], highlighting the complexity of achieving long-lasting treatment effects in inherited retinal diseases.

#### 2.4.2. Lysosomal Storage Disorders

Lysosomal storage disorders, rare metabolic conditions caused by deficiencies in lysosomal proteins and enzymes, lead to the accumulation of substances within various organs [113,114,115]. These disorders have multi-organ presentations and often involve the central nervous system (CNS), leading to severe and progressive neurodegeneration that significantly impacts the prognosis. Traditional therapies, including enzyme replacement therapy, have limited efficacy in targeting CNS manifestations due to the BBB. Gene therapy offers a promising solution, particularly in developing replacement therapies for intracellular enzymes. Currently, around 40 clinical trials are registered using gene therapy in LSDs, including long-term follow-up studies. For treating CNS manifestations in LSDs, in vivo gene therapy employing AAVs has become a leading strategy [116]. These can be administered directly to the brain or cerebrospinal fluid (CSF) through various methods, including intracerebral, intrathecal, intracerebroventricular, or intracisternal routes [117]. AAV serotypes like AAV9 and AAVrh10, known for their CNS targeting and ability to cross the BBB, are commonly used in clinical settings [117,118,119]. Since lysosomal disorders present with a series of neuro-metabolic pathologies, using a systemic administration approach using AAV9 could be beneficial to treat both the visceral organs and the CNS with one single dosing. Innovative AAV serotypes engineered for an enhanced CNS tropism after intravenous administration are also being explored [33].

#### 2.4.3. Neurological Disorders

The first FDA-approved AAV gene therapy for a neurological disorder was Zolgensma, an AAV9-based gene therapy for type I spinal muscular atrophy (SMA), administered to pediatric patients via a single intravenous dose [120,121]. In its clinical trial, all of the first 15 treated patients remained event-free at 20 months of age, a significant improvement compared to the 8% survival rate observed in a historical cohort [121]. Two deaths were reported during the phase III studies; however, the respiratory complications were not deemed to be related to the therapeutic intervention [122]. The postmortem analysis of the patients allowed researchers to demonstrate, for the first time, the widespread biodistribution of the AAV9 vector throughout the CNS and peripheral organs following systemic delivery in humans. This success has spurred interest in applying gene therapy to other genetic neurological diseases, with multiple ongoing clinical trials exploring this approach. For monogenic recessive diseases, the target for gene therapy is the mutated gene itself. However, for complex diseases like Parkinson’s and Alzheimer’s, the focus may be on a protein that could enhance neurological functions or the primary genetic cause [21]. AAV9 is currently the preferred vector for broad CNS transduction, although studies have also utilized capsids such as AAV2, AAV6, AAV9, AAVrh10, and AAVhu [123,124]. For targeting specific brain regions, such as in Parkinson’s disease, AAV2 is often chosen due to its well-documented spread and tropism, as well as its relatively limited spread beyond the injection site [125]. The route of administration is crucial for the biodistribution of gene expression, with several methods being explored in ongoing trials to optimize delivery and therapeutic outcomes. These include intravenous administration that circulates the gene therapy body-wide, intraparenchymal administration (IPa) that targets specific brain areas, and intracerebrospinal fluid (intra-CSF) routes that deliver the AAVs within the CNS [21].

#### 2.4.4. Blood Disorders

Blood disorders, particularly hemophilia A and B, are characterized by deficiencies in coagulation factors VIII (FVIII) and IX (FIX), respectively [126]. These deficiencies impair blood clotting, leading to severe bleeding episodes. AAV gene therapy represents a promising approach for treating these disorders. It involves a single-dose, intravenous administration aimed at the long-term continuous endogenous synthesis and secretion of the missing coagulation factor to reduce bleeding risks. This approach uses recombinant AAV vectors targeting hepatocytes that deliver FVIII or FIX variant transgenes with liver-specific promoters [127].

Hemophilia A is the subject of the most clinical trials, particularly those in late-phase development, indicating a higher likelihood of commercialization. In 2023, the FDA approved Roctavian, a leading gene therapy for adults with severe hemophilia A who do not have pre-existing antibodies against AAV serotype 5. In November 2022, the FDA approved Hemgenix for patients with hemophilia B (congenital factor IX deficiency), followed by its approval of Bevqez in April 2024 for the treatment of adults with moderate to severe hemophilia B, marking Pfizer’s first regulatory approval for this type of gene therapy. Various phase I/II clinical trials in the US and Europe have tested AAV vectors (AAV2, AAV5, AAV6, AAV8, the mutant AAV Spark100, and AAV-LK03) in patients with hemophilia A and B at different doses. These trials have demonstrated increases in the clotting factor activity ranging from 5% to 400%. However, some patients experienced elevated liver enzymes 6–10 weeks post-administration, indicating potential liver-related side effects [43,128,129,130,131]. The use of a modified FVIII has led to a more efficacious therapy, with a decreased dose requirement, increased durability due to the more stable expression of the factor, and reduced intracellular stress.

#### 2.4.5. Muscular Diseases

Duchenne muscular dystrophy (DMD) results from the lack of dystrophin, a crucial protein for maintaining the integrity of muscle cells. The absence of dystrophin leads to progressive muscle weakness and deterioration. The FDA’s approval of Elevidys marked the first gene therapy for treating pediatric patients aged 4 to 5 with DMD, representing a significant advancement in DMD treatment, which previously had limited options focused primarily on symptom management. Elevidys works by delivering an AAV rh74 vector that produces a modified version of the dystrophin protein, which is absent in individuals with DMD. Administered via a single intravenous dose, Elevidys has shown an increased expression of micro-dystrophin in treated patients, thereby helping to maintain the muscle cell integrity.

Other clinical studies are ongoing with various AAV serotypes (AAV1, AAV8, AAV9) and transgenes (encoding mini-dystrophin, micro-dystrophin, follistatin, and β-1,4-N-acetylgalactosaminyltransferase 2) to treat Duchenne muscular dystrophy and with the MTM1 transgene to treat patients with X-linked myotubular myopathy [132,133].

### 2.5. Advancements in Gene Therapy for Aging

**Figure 4:**
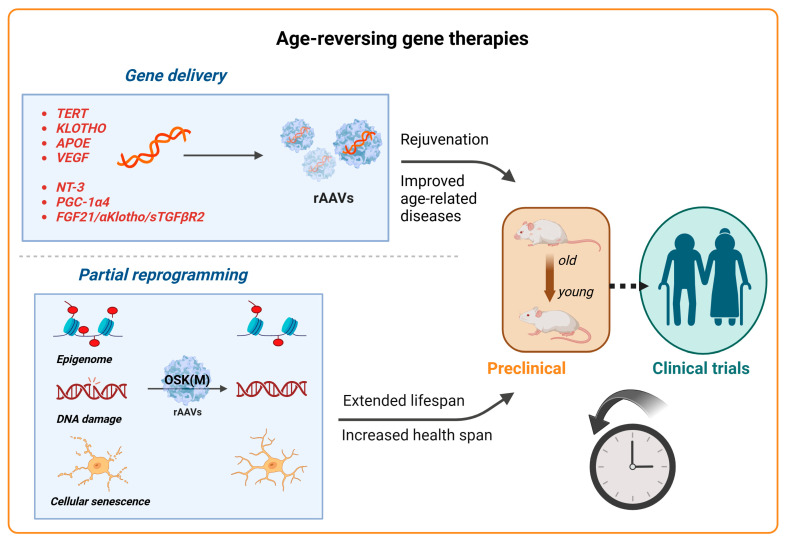
Advancements in gene therapy for aging. Schematic representation of recent advancements in gene therapy targeting aging and age-related diseases. Top panel: AAV vectors for the delivery of therapeutic genes are being explored for various aging-related targets, for example, *TERT* to extend the lifespan and *klotho* to treat age-related diseases and improve cognition. *APOE* (for Alzheimer’s) and *VEGF* (for vascular health) are also under investigation. The AAV-mediated delivery of PGC-1α4 and NT-3 has demonstrated the restoration of muscle function in sarcopenia in mouse models, while AAV8-based therapies targeting longevity-associated genes (FGF21, α-klotho, and sTGFβR2) have enhanced the health span and longevity in multiple age-related disease models. Bottom panel: Emerging strategies like partial reprogramming, using the AAV-mediated delivery of Yamanaka factors (Oct4, Sox2, Klf4, and c-Myc; OSKM), aim to rejuvenate cells, tissues, and organisms, showing the potential to reverse aging-related symptoms and improve longevity. Gene therapy approaches for targeting age-related diseases and phenotypes are still largely in the preclinical stages (animal models and early testing), with clinical trials expected to follow.

Aging is a natural and complex process marked by a progressive decline in physiological functions, making it a principal risk factor for many diseases, including neurodegenerative disorders, cardiovascular and metabolic diseases, and cancers. It is characterized by the chronic dysregulation of cellular processes, leading to dysfunction at the cellular, tissue, and organ levels [134]. Key aspects of aging involve primary hallmarks such as genomic instability, telomere attrition, and the loss of proteostasis, alongside antagonistic hallmarks such as mitochondrial dysfunction and cellular senescence, culminating in stem cell exhaustion and impaired intercellular communication [134]. While aging itself cannot be prevented, interventions aimed at optimizing cellular functions offer a pathway to enhance both the lifespan and health span. Despite the rapid advancements in clinical trials for AAV treatments for rare diseases, gene therapy approaches for treating complex aging or age-related diseases are still in their preclinical stages. Here, we review the current advancements in anti-aging AAV-based gene therapies that target age-related genes, age-associated diseases, and the emerging field of partial reprogramming (Figure 4). These approaches hold significant potential not only to improve the individual quality of life but also to reduce the socioeconomic burdens associated with aging.

#### 2.5.1. Aging-Related Genes

*TERT*: Research into telomere dysfunction, which is linked to aging through the process of telomere shortening with each cell division, has prompted the exploration of the telomerase reverse transcriptase (*TERT*) gene—a vital component of telomerase—as a potential therapeutic target [135]. Several studies have indicated that *TERT* gene therapy is a promising strategy to delay aging and increase longevity without elevating the risk of cancer. Initially, Bernardes de Jesus et al. demonstrated that delivering an AAV–mouse *TERT* into mice aged 12 and 24 months resulted in significant enhancements in aging-associated molecular biomarkers (such as cyclin D1 and β-catenin), the reactivation of telomerase activity, and an increased median lifespan compared to a control group with inactive *TERT* [136]. Subsequently, Bär et al. created an AAV9 vector to express *TERT*, targeting heart failure post-myocardial infarction. This intervention led to reduced cardiac damage, lower mortality rates, and improved health indicators in treated mice [137]. Further research showed that high doses of this AAV9-*TERT* vector effectively treated aplastic anemia in mice with short telomeres, enhancing bone marrow function, the telomere length, and survival rates [138]. Povedano et al. then applied this therapy in a mouse model of pulmonary fibrosis induced by low-dose bleomycin [139], and Whittemore et al. addressed neurodegeneration related to short telomeres, with both studies observing significant improvements [140]. Transitioning from preclinical research to clinical application, researchers have launched human trials using an AAV-*hTERT* vector, LGT, targeting aging (NCT04133649), Alzheimer’s disease (NCT04133454), and critical limb ischemia (NCT04110964) through three separate phase I trials initiated in 2019 [2]. These clinical trials, utilizing AAV vectors to deliver *TERT* gene therapy, are focused on combating aging and its related diseases, representing a crucial step in translating this research into human health benefits.

*Klotho (KL)*, a molecule with anti-aging properties, has shown promise in its potential to slow down and possibly reverse the effects of aging [141,142]. *KL* plays a critical role in pathways linked to chronic age-related conditions, including kidney disease, tissue degeneration, and neurodegenerative diseases [143,144]. Preclinical studies have demonstrated *KL*’s substantial promise in tackling a broad spectrum of aging-associated diseases. For instance, a systemic increase in the secreted α-klotho hormone has shown an improvement in the synaptic plasticity and cognitive function in mice [145]. While *KL* is mainly known for its involvement in cognitive functions in aging, this factor is implicated in a series of other cellular processes, including insulin regulation and cell growth in cancer [145]. Generally, the *KL* promoter is hypermethylated in tumor tissues, with a consequent decrease in the *KL* expression [146]. *KL* has been linked to the inhibition of insulin and insulin-like growth factor 1 (IGF-1) pathways, which regulate cell proliferation and breast cancer tumorigenesis [147]. *KL* is mainly expressed in the renal tubules and the brain; however, it is also found in sex hormone-responsive organs, such as the placenta and ovaries. Low *KL* expression is reported in ductal carcinoma and other invasive forms of breast cancer. The normalization of *KL* expression in breast cancer cells reduces tumor growth, activates tumor suppressor elements, and upregulates the fibroblast growth factor (FGF) signaling pathway, reducing the overall clonogenic proliferation. *KL* also acts as a Wnt antagonist [148], contributing to the reduction in stem cell depletion during aging, and binds to the FGF23 receptor, involved in phosphate homeostasis in the kidney [149]. In progressive chronic kidney disease, increased levels of circulating FGF23 and low *KL* expression in the kidney lead to severe cardiovascular manifestations, including heart failure and ultimately death. Increased levels of KL have been shown to mitigate cardiovascular complications [150]. *KL*’s antineoplastic effects in breast, lung, cervical, and thyroid cancer [151] also suggest it could be a valuable therapeutic target with potential applications in gene therapy to improve longevity while treating multiple age-associated disorders and malignancies [152]. Since *KL* does not cross the BBB, the systemic delivery of gene therapy vectors to increase KL levels is a promising approach for these conditions.

One gene therapy strategy involves using an AAV-FGF21 vector to enhance the interaction between the fibroblastic growth factor FGF21 and its coreceptor β-klotho, thereby activating the ERK1/2 and MAPK signaling pathways to address metabolic diseases [153,154]. Another approach utilizes AAV9 vectors for the intraventricular delivery of klotho isoforms into senescence-accelerated mouse prone 8 (SAMP8) models. Overall, AAV9-mediated *KL* expression has shown promising anti-aging effects, improving both molecular and physiological aspects of aging in SAMP8 mice [155].

While gene therapy is a promising tool to deliver *KL*-based treatment, sustained expression levels are needed for a therapeutic benefit in patients with renal and cardiovascular dysfunctions where gene expression is downregulated and *KL* production is impaired [156]. However, since *KL* expression is generally low in most tissues, off-target overexpression must be carefully evaluated to ensure its safety. While currently there is no indication suggesting that maintaining physiological or supraphysiological levels of *KL* is linked to adverse side effects, high *KL* levels can lead to hypotension, hypophosphatemia, and insulin resistance [146]. Therefore, the effect of prolonged and widespread *KL* expression should be evaluated, together with a possible interaction with standard cancer treatment regimes.

*APOE*: The *APOE* gene is closely linked to human longevity and plays a crucial role in lipid metabolism, with significant variations affecting the lifespan and Alzheimer’s disease (AD) risk [157]. There are three predominant *APOE* alleles in humans, which confer different levels of risk for developing AD and related dementias. The *APOE4* allele is associated with reduced longevity and a higher risk of sporadic and late-onset AD, with a 15-fold increased risk in homozygotes, while *APOE2* reduces the AD risk by almost half and tends to be protective against these conditions [158]. Research efforts include modifying the *APOE* expression and function in mouse models, with some studies attempting to switch the *APOE* alleles through gene therapy for therapeutic benefits. A pivotal clinical trial (NCT03634007) has experimented with using AAV vectors to convert *APOE4* to *APOE2*, aiming to mitigate AD’s impact [2]. Ongoing studies focus on unraveling *APOE*’s mechanisms and its broader therapeutic potential [159].

*VEGF*: Vascular endothelial growth factor (*VEGF*) is crucial for maintaining blood vessel networks, whose aging can significantly affect the health of the entire organism [160]. A study by Grunewald et al. suggested that enhancing *VEGF* signaling in mice, achieved through genetic modification or AAV-*VEGF* delivery, mitigated the effects of aging on blood vessels, improving tissue oxygenation and the blood flow and addressing signs of aging such as mitochondrial dysfunction and inflammation. This approach not only extended the lifespan of mice but also improved their health span by reducing symptoms of aging like fat accumulation, liver dysfunction, muscle and bone loss, and the incidence of spontaneous tumors, thereby offering a broad protective strategy against age-related deterioration [161]. While preclinical studies have shown that the overexpression of *VEGF* in the heart might cause an inflammatory response in larger animal models [162], further research into the optimal vector, dose, and route of administration is needed.

#### 2.5.2. Age-Related Diseases

Sarcopenia, a condition characterized by the progressive loss of skeletal muscle mass and strength during aging, poses significant health risks for the elderly, leading to increased falls, fractures, and consequent mortality [163,164]. Its onset typically begins after the age of 40, escalating significantly beyond the age of 70 and affecting up to half of those over 80 [165,166]. While traditional approaches to prevent or manage this condition focus on exercise and nutrition, limitations due to diet, malabsorption, or mobility issues necessitate innovative solutions [167]. Advances in gene therapy offer promising avenues for mimicking the muscle-preserving effects of exercise, targeting the molecular underpinnings of muscle atrophy. Guo et al. introduced a novel approach to treat sarcopenia by engineering a nuclear-targeted form of PGC-1α4 (*NLS-PGC1α4*) and delivering it into mouse muscles using an AAV vector. This intervention led to an increased muscle size and strength and improved metabolic health markers [168]. In another study, Ozes et al. demonstrated the efficacy of neurotrophin 3 (*NT-3*) gene therapy in addressing sarcopenia and associated aging effects. By employing a muscle-specific creatine kinase promoter and a self-complementary AAV (scAAV1) for *NT-3* delivery, the therapy resulted in aged C57BL/6J mice showing an enhanced muscle fiber diameter. This was shown alongside improvements in the neuromuscular junction connectivity and anti-inflammatory, antioxidant, and antiapoptotic effects, as well as increased mitochondrial biogenesis [169]. Overall, these approaches, leveraging AAVs for precise delivery, offer a promising therapeutic strategy against sarcopenia, showcasing the potential to restore muscle function and systemic energy metabolism in the elderly.

Multiple diseases: Traditional approaches to disease treatment often target single diseases, which overlooks the interconnected nature of age-related conditions, leading to inefficient treatments with the potential for compounded side effects. A study by Davidsohn et al. explored the development of gene therapies aimed at simultaneously treating multiple age-related diseases to improve the health span and longevity. By using three liver-targeted AAV8-based gene therapies targeting longevity-associated genes (fibroblast growth factor 21, FGF21; α-klotho; and the soluble form of mouse transforming growth factor-β receptor 2, sTGFβR2), this research aimed to tackle the combined pathologies of four age-related diseases, obesity, type II diabetes, heart failure, and renal failure, with a single treatment strategy. The study found that individual and combined applications of these gene therapies could effectively treat and, in some cases, reverse these conditions in mouse models. For instance, a notable outcome was a 58% increase in heart function in models of heart failure and a complete reversal of obesity and diabetes phenotypes in models fed a high-fat diet. Furthermore, a single formulation combining two separate therapies (AAV:sTGFβR2 and AAV:FGF21) was capable of addressing all four diseases simultaneously, showcasing the potential of combination gene therapy to effectively improve the health span. This research is therefore paving the way for future treatments that could enhance both longevity and the quality of life [170].

#### 2.5.3. Partial Reprogramming

Partial reprogramming is an emerging approach aimed at rejuvenating cells, tissues, or even whole organisms, potentially reversing aspects of aging and improving health and longevity. Multiple studies have shown that the transient expression of the Yamanaka factors Oct3/4, Sox2, KLF4, and c-Myc (OSKM) can mitigate age-related symptoms and revert aged cells to a more youthful state without losing their full epigenetic identity, thus avoiding the completion of the full reprogramming cycle [171,172,173,174,175,176,177]. However, the expression of c-Myc may increase the risk of developing teratomas [178,179,180,181]. Therefore, recent efforts focusing on the delivery of only three reprogramming factors (OSK, without c-Myc) have substantially increased the safety and efficacy of partial reprogramming while reversing signs of aging and degeneration in CNS tissues, specifically in the context of glaucoma and other age-associated conditions. For example, Lu et al. demonstrated that the ectopic expression of an AAV2-tTA and TRE-OSK-inducible system in mouse retinal ganglion cells not only restored youthful DNA methylation patterns and transcriptomes but also promoted axon regeneration after injury and reversed vision loss in models of glaucoma and in aged mice [174]. In a follow-up year-long study, Karg et al. showed that continuous or cyclic AAV-OSK expression resulted in a remarkable restoration of vision, with effects lasting 11 months post-treatment. This study emphasized the therapeutic potential of OSK-based rejuvenation for glaucoma, underscoring its efficacy and safety even with prolonged expression (up to 21 months) [182]. Recently, Macip et al. further demonstrated that the systemic expression of AAV9 encoding an inducible OSK system not only extended the median remaining lifespan of 124-week-old male mice by 109% over wild-type controls but also significantly improved health parameters and frailty scores, indicating an enhancement in both the lifespan and health span [183]. Overall, the safety and efficacy demonstrated in these interventions, particularly with prolonged treatment durations, highlight the therapeutic potential of epigenetic rejuvenation AAV-based therapies not only in extending the lifespan but also in improving the health span and quality of life in aged populations.

### 2.6. Challenges in AAV Gene Therapy

Despite considerable progress in the field of AAV gene therapy, several persistent challenges hinder its transition from preclinical models to effective clinical applications. These challenges are primarily rooted in the unique biological properties and interaction dynamics of AAV vectors with the human immune system, as well as technical limitations in vector design and delivery, discrepancies between animal models and human clinical outcomes, and the need for high vector doses.

(i) Vector delivery limitations: One of the foremost challenges is the limited gene packaging capacity of AAV vectors (approximately 4.7 kb), which restricts the size of the therapeutic genes they can carry and thus limits their use in treating diseases caused by larger genes. To overcome this limitation, strategies such as engineering ‘mini gene’ constructs or using dual or triple AAV vectors have been developed. These involve splitting large genes into smaller segments that can be delivered by multiple AAV vectors [184]. Several studies in animal models have confirmed the effectiveness of the split strategy in delivering larger genes into host cells through the use of dual or triple AAV vectors [185,186,187,188]. Nevertheless, this approach raises challenges in manufacturing, obtaining regulatory approval, and ensuring consistent therapeutic efficacy. Additionally, potential immune responses due to the higher capsid protein burden and the high cost of development and deployment also pose significant challenges. Another limitation of AAV delivery is the possibility of off-target transduction, where vectors infect cells other than their intended targets, which can lead to unintended effects and reduce the efficiency and durability of gene therapy [97]. Moving forward, exploring new and more tissue-specific and endogenous promoters is desirable to achieve high and sustained transgene levels in humans.
(ii) Immunogenicity: A critical barrier, often unaddressed until the clinical stages, is the immune response elicited by both the AAV capsid and the transgene product. While AAV vectors are not considered a strong elicitor of an innate or adaptive immune response, preclinical and clinical findings have demonstrated that capsids, genomes, and gene products can elicit a response. Humoral and cell-mediated responses can occur following AAV dosing, particularly when the therapy is administered systemically and at high doses. The immune response includes pre-existing antibodies to AAV capsids, limiting the transduction and efficacy. A large segment of the population has pre-existing adaptive immunity against wild-type AAVs, including neutralizing antibodies (NAbs) and T cells that can lead to the loss of transgene expression or the elimination of transduced cells [189,190]. In addition, cellular stress from high levels of transgene expression can initiate the apoptotic signaling cascade, leading to cell death. In particular, for conditions like hemophilia, up to 40% of adult patients may be ineligible for liver-directed AAV trials due to pre-existing NAbs and memory T cells directed against the AAV capsid proteins [191]. Even if NAbs are initially absent, they can develop rapidly after exposure to the vectors, hindering repeated administration and the overall treatment efficacy. This pre-existing immunity not only reduces the clinical efficacy but also prevents the possibility of re-administration and significantly limits the patient eligibility for clinical trials among a large proportion of the general population [11,192,193,194]. This poses an obvious question on the need for repeated dosing to achieve a therapeutic benefit in the long term, particularly in childhood patients, where the benefit of the therapy could diminish over time due to a reduction in gene expression. To address these challenges, novel strategies are being developed: the sequential administration of different capsids with low cross-reactivity, engineering mutations into the AAV capsid that can prevent NAbs from binding, the conjugation of AAV particles with biological extracellular vesicles [195], and evolving AAV libraries in an in vitro environment full of Nabs, which can create neutralization-resistant vectors [196,197]. Additional techniques, such as pharmacological treatments to degrade circulating IgG, plasmapheresis, and B cell depletion or reduced activation via the administration of rapamycin have shown promise in reducing anti-AAV immune responses [198,199,200,201]. These approaches offer different prospects and challenges and are in various stages of development requiring extensive preclinical testing and validation before they can be widely implemented in clinical settings. In addition, localized administration to immune-privileged organs such as the eye or the brain could be a valid mitigation strategy for clinical application. The route of administration influences the risk of immunogenicity and the therapeutic efficacy of the treatment, as different organs and tissues present a different immune environment and number of antigen-presenting cells. Since an immune response can arise at any stage of development and treatment, comprehensive risk assessments are needed to evaluate and identify possible risk factors throughout the clinical studies, either related to the product itself (CpG content, serotype, tropism), the manufacturing process (impurities, biosynthetic intermediates), or the patient (pre-existing antibodies, treatment eligibility and regime). Finally, empty capsids can increase the exposure of patients to the AAV load, contributing to a potential immune response and, therefore, hindering the clinical outcome. For example, empty AAV8 capsids can trigger strong B and T cell responses in humans and inhibit the transduction of hepatocytes in mice [202]. Therefore, a low empty capsid content will increase the ratio of therapeutic DNA per mass of the capsid protein, improving the safety profile of the treatment. The safety risk assessment of gene therapies also includes the analysis of the integrity of the genetic material, including the insertional mutagenesis risk, risk of germline transmission, and possible off-target effects in genome editing studies.
(iii) Translational hurdles: Significant challenges in translating AAV gene therapy stem from discrepancies between animal models and human clinical outcomes. Animal studies often fail to predict human immune responses accurately, leading to unforeseen adverse effects in clinical settings. This issue is compounded by species-specific differences in the vector tropism, which can alter the efficiency of gene transfer between preclinical and clinical settings [203]. These differences in the vector tropism are partly due to the varied expression of cellular receptors. In addition, differences in how the virus is processed within cells post-entry, such as nuclear translocation and the conversion of the genome from single-stranded to double-stranded, also play a significant role. These complexities raise critical questions about the accuracy of animal models in predicting human outcomes and underscore the need for targeted human-based studies to ensure successful clinical translations. Biodistribution and vector processing studies are usually conducted in the preclinical setting, with only limited data in humans, while a more comprehensive understanding of viral shedding is available from clinical trials. Biodistribution studies are required to evaluate the relationship between efficacy and safety outcomes and the exposure of target and non-target tissues to the product. While stand-alone biodistribution data are not always required for early product development, preclinical shedding data are requested before starting the first in-human dosing. A summary of the global guideline documents on biodistribution and shedding is presented in Table 5.

Based on the FDA guidance, the minimum panel of tissue collection in a preclinical GLP-compliant biodistribution study should include the liver, spleen, kidney, lung, heart, brain, gonads, blood, and possibly the injection site. Additional organs might be required for specific routes of administration, e.g., the spinal cord for CSF delivery. An analysis of the kinetics of vector distribution, gene expression, and any clinical pathology can determine whether the treatment correlates with any adverse finding. The vector copy number and transgene expression are the main readouts for biodistribution and toxicology studies. When the native protein product cannot be distinguished from the vector-encoded product, the characterization of mRNA expression is used to identify efficacy endpoints.

(iv) Dosing regimen: The high vector doses necessary for effective treatment, particularly for multi-systemic indications, have been associated with severe immune reactions such as hepatotoxicity, complement activation, and even acute kidney injury in some cases. A T cell-mediated immune response to high levels of capsid antigens has emerged as the most common adverse effect [4]. Severe adverse events have been reported in various clinical trials involving high AAV vector doses (6.7 × 10^13^–3 × 10^14^ vg/kg body weight) administered to neonates and children. These include complement activation and acute kidney injury in the Duchenne muscular dystrophy trial, elevated serum transaminase levels in the SMA1 Novartis trial, and a fatal case in the ASPIRO trial [97,204]. To mitigate such risks, future research should prioritize developing optimal dosing regimens that balance toxicity and efficacy, particularly through studies in non-human primates. Dose selection can be particularly complex, as it is based on both efficacy and safety, and the pharmacokinetic/pharmacodynamic properties of AAVs are heavily affected by their biological fate after administration. The No-Adverse Event Level (NOAEL) dose is determined in preclinical studies and is usually used as the upper limit of the dose range when scaling from animals to humans. However, due to the physiological differences between species, animal models might not fully recapitulate the effects in humans. Therefore, the maximum tolerated dose should be based not only on the preclinical NOAEL but also on historical clinical data from trials using the same capsid and route of administration. A positive risk/benefit assessment is essential, particularly if the target population is largely pediatric and if the route of administration requires invasive surgical procedures. The pharmacologically active dose at multiple endpoints should be carefully calculated prior to the first in-human dosing.

A general dose scaling equation can be used to estimate the translation from animals to humans:

dose_H_ = dose_A_ × transduction factor × (metric_H_/metric_A_)

with the metrics including either the body weight for intravenously administered AAVs or the organ mass or volume for localized administration.

For Zolgensma, the dose scaling was based on the efficacy studies in the SMA mouse model, using the total body weight as a morphological factor, with a transduction factor of one (US FDA 2019 ZOLGESNMA highlights the prescribing information).

(v) Safety concerns and biomarker assessment: Preclinical safety profiles and clinical pharmacology data indicate that AAV gene therapy is efficacious overall and well tolerated. However, in recent years an association between AAV gene therapy and specific safety findings, particularly hepatotoxicity and thrombotic microangiopathy, has been reported, with some treatments leading to serious adverse events and the death of study subjects. Other toxicity events, such as dorsal root ganglia and peripheral nerve toxicity, have been reported only in animal models. The assessment of biomarkers is essential to evaluate pathogenic processes and the response to the therapeutic intervention. This is of particular importance to assess the genotoxicity, immune-mediated toxicity, study enrolment, and patients’ stratification (fda.com/biomarker-qualification-program). Substrates and downstream metabolites, assessed via mass spectrometry, histology, or imaging techniques, are usually the primary biomarkers that can indicate disease pathogenesis and progression.

Table 6 outlines the major challenges in AAV gene therapy described above, and proposed solutions are outlined.

## 3. Conclusions and Future Prospects

AAV vectors, known for their safety, low immunogenicity, and ability to deliver sustained transgene expression, have become a pivotal tool in gene therapy. Their capacity for long-lasting transgene expression in various types of cells, broad tissue tropism, high transduction efficiency, and strong safety record underscore their widespread use in both clinical and preclinical settings. These vectors have facilitated remarkable clinical successes across diverse therapeutic areas, including retinal diseases, hemophilia, and neuromuscular disorders, demonstrating their versatility and effectiveness. The recent FDA approval of six AAV-based therapies is evidence of their potential to significantly improve patient care and treatment outcomes. Additionally, the potential of gene therapy to address aging and age-related diseases presents an exciting frontier, although it requires further exploration and development. The manufacturing process for AAV vectors continues to evolve, with advances aimed at enhancing the scalability and efficiency to meet the growing demand for these therapies.

Looking forward, the future of AAV gene therapy appears promising, though overcoming several challenges is necessary to fully realize its potential. While the technology has matured, critical hurdles remain, including immune response management, vector delivery optimization, the scalability of production processes, and cost reduction. Continued efforts in vector engineering, particularly the development of innovative capsid designs, are crucial for evading the immune system and improving gene delivery. For instance, advances in structure-guided evolution and machine learning have enabled the creation of de-immunized capsids that reduce immunogenicity while maintaining high transduction efficiency [205]. These advancements are exemplified by efforts to treat conditions such as muscular dystrophy [206] and hemophilia [130], where targeted delivery and enhanced immune evasion are crucial for successful outcomes. Another emerging approach to mitigating immune responses involves combinatorial AAV therapies, where multiple AAV vectors deliver different therapeutic genes or components to target cells [207]. Similarly, combinatorial strategies could synergistically target multiple pathways, such as delivering both a therapeutic gene and a regulatory RNA for neurodegenerative or cardiovascular diseases. These approaches offer a platform to expand the AAV applicability to more intricate genetic and multi-system disorders.

Emerging technologies like CRISPR and other genome-editing tools are also being integrated with AAV systems. AAV vectors are increasingly used to deliver CRISPR-Cas9 nucleases and donor DNA constructs for precise genome editing. This approach allows for targeted modifications and the insertion of therapeutic genes at specific chromosomal locations [208]. Combining AAVs with CRISPR-Cas9 technology, which has already shown preclinical success [209], could open new avenues for treating complex genetic diseases more effectively. This strategy enhances the efficacy of gene editing while improving the overall accuracy and safety.

Bridging the gap between preclinical research and clinical application is another critical challenge for advancing AAV-based therapies. Studies involving non-human primates have provided valuable insights into the long-term expression and safety profiles of gene therapies, informing the design of clinical trials. Additionally, identifying reliable biomarkers to monitor the efficacy and immune responses during trials is expected to streamline clinical development timelines. Combining gene therapy with cell therapy offers another powerful approach to treating complex diseases. For example, AAV vectors can deliver therapeutic genes to stem cells or immune cells to enhance their therapeutic potential. This combined approach is being explored for various conditions, including genetic disorders, cancers, and immune deficiencies. Finally, continued research, collaborative efforts between academia and industry, and supportive regulatory frameworks will be essential to navigate the complexities of gene therapy development. Harnessing the transformative potential of AAV technologies will require a multidisciplinary effort to ensure their safe and effective application in clinical settings.

## Figures and Tables

**Table 1:** Summary of AAV-mediated delivery methods.

Delivery Method	Target Tissue/Organ	Advantages	Limitations
Intravenous (IV)	Liver, muscle, central nervous system (CNS), systemic tissues	Non-invasive Broad distribution to multiple organs Suitable for systemic disorders	Risk of immune response Potential off-target effects Limited targeting specificity
Intrathecal (IT)	Central nervous system (spinal cord, brain)	Direct delivery to CNS Bypasses blood–brain barrier Lower systemic exposure	Requires expertise Invasive May need repeated administration
Intramuscular (IM)	Skeletal muscle	Localized and sustained expression Minimally invasive Easy administration	Limited to muscle-targeted applications Risk of immune response at injection site
Intraperitoneal (IP)	Abdominal organs (e.g., liver, pancreas)	Localized delivery to abdominal cavity Minimally invasive	Limited applicability for systemic diseases Risk of peritoneal adhesions
Intraocular	Retina	Localized delivery Bypasses systemic circulation Direct access to target tissue	Invasive Limited to ocular applications Risk of complications such as retinal detachment

**Table 2:** Comparative overview of AAV serotypes.

AAV Serotype	Tropism	Transduction Efficiency	Immune Response	Clinical Applications
AAV1	Skeletal muscle, heart, CNS, retina	High for skeletal muscle and cardiac tissues	Moderate humoral immune response; reduced immune recognition due to lack of heparin binding	Gene therapy for muscular and cardiovascular disorders, ocular therapies
AAV2	Liver, retina, CNS, skeletal muscles	High; recognized for its versatility in multiple tissues	High pre-existing immunity in humans	Widely used in research and clinical trials, including for Parkinson’s disease and blindness caused by Leber congenital amaurosis, hemophilia B, and CNS-related therapies
AAV3	Liver, hepatic cancer cells	High for human hepatocytes; low in murine cells	Low	Emerging applications in liver-targeted therapies
AAV4	Retina, CNS	Moderate for CNS and retinal cells	Low	Ocular gene therapy, CNS-related therapies, and retinal applications
AAV5	Airway epithelia, liver, murine retinal cells, neural cells (Purkinje, basket neurons)	High for retinal and neural tissues	High	Retinal gene therapy, airway-related diseases, and CNS therapies
AAV6	Skeletal muscle, liver, airway epithelia	High for skeletal muscles and cardiac tissues	Moderate	Respiratory disease therapies and muscle-targeted treatments
AAV7	Skeletal muscle, liver, CNS, retina	High in muscle tissues and hepatocytes	Low	CNS-related applications, retinal gene therapy
AAV8	Liver, skeletal and cardiac muscles	Highest efficiency for liver and muscle cells	Low immune response compared to other serotypes	Liver-directed gene therapies, retinal disorders, and muscular dystrophies
AAV9	CNS, heart, skeletal muscle, liver, retina	High for CNS and peripheral tissues	Low to moderate; capable of crossing BBB	Neurological disorders (e.g., SMA), cardiac gene therapies
AAV10	Intestines, liver, lymph nodes, kidneys, adrenal glands, retina	High for a broad range of tissues	Moderate	Emerging gene therapy applications, including for intestinal and retinal disorders
AAV11	CNS, murine projection neurons and astrocytes	Moderate	Moderate; less studied	CNS-targeted therapies
AAV12	Salivary glands, nasal epithelia	High	Low	Respiratory gene therapy and experimental applications

**Table 3:** Comparison of AAV purification techniques.

Purification Technique	Purity	Yield	Scalability	Cost	Clinical Grade Compliance
Affinity Chromatography	High (>98% purity), but does not separate empty and full capsids	Stable yield across batches	Scalable with automated processes; requires specific resins	Moderate to high due to resin costs and regeneration needs	GMP-compatible; suitable for large-scale production
Ion Exchange Chromatography (IEC)	High (>98% purity); removes empty capsids effectively	High and consistent yields	Scalable with automated systems; resin lifetime may vary	Moderate due to resin limitations and process complexity	GMP-compatible; effective for clinical-grade vectors
Ultracentrifugation	High purity; effective for separating full from empty capsids	Lower yields compared to chromatography	Limited scalability due to time and equipment constraints	Low for small scale, high for larger scale due to labor and equipment	Challenging to scale; not ideal for GMP compliance
Tangential Flow Filtration (TFF)	Complements other techniques, achieving >90% full particles	Supports concentration and buffer exchange; retains high yield	Scalable and suitable for large volumes	Cost-effective for downstream steps	GMP-compatible when integrated into purification workflows

**Table 4:** FDA-approved AAV gene therapies.

Product Name	Year of Approval	Indication	Vector Type	Manufacturer
BEQVEZ (fidanacogene elaparvovec-dzkt)	2024	Adult hemophilia B	rAAVrh74var	Sarepta Therapeutics, Inc. Cambridge, MA, USA
Elevidys (delandistrogene moxeparvovec-rokl)	2023	Duchenne muscular dystrophy	rAAVrh74	Sarepta Therapeutics, Inc. Cambridge, MA, USA
Hemgenix (etranacogene dezaparvovec-drlb)	2022	Adult hemophilia B	rAAV5	CSL Behring LLC. King of Prussia, PA, USA
Luxturna (voretigene neparvovec-rzyl)	2017	Biallelic RPE65 mutation-associated Leber congenital amaurosis	rAAV2	Spark Therapeutics, Inc. Cambridge, MA, USA
Roctavian (valoctocogene roxaparvovec-rvox)	2023	Adult hemophilia A	rAAV5	BioMarin Pharmaceutical, Inc. San Rafael, CA, USA
Zolgensma (onasemnogene abeparvovec-xioi)	2019	Spinal muscular atrophy type I	rAAV9	Novartis Gene Therapies, Inc. Bannockburn, IL, USA

**Table 5:** International regulatory guidance documents.

Document	Committee
Guideline S12 on non-clinical biodistribution considerations for gene therapy product (2023)	ICH
Reflection paper on expectations for biodistribution assessment for gene therapy product (2018)	IPRP
Long-term follow-up after administration of human gene therapy products (2020)	FDA
Guideline on the quality, nonclinical, and clinical aspects of gene therapy medicinal products (2018)	EMA

ICH: International Council for Harmonisation of Technical Requirements for Pharmaceuticals for Human Use; IPRP: International Pharmaceutical Regulators Programme; FDA: Food and Drug Administration; EMA: European Medicines Agency.

**Table 6:** Major challenges in AAV gene therapy and proposed solutions.

**Challenge**	**Description**	**Proposed Solutions**
Limited Packaging Capacity	AAV vectors can carry only ~4.7 kb, restricting their use for larger therapeutic genes	Use of ‘mini gene’ constructs Dual or triple AAV vectors to deliver split genes Advanced capsid engineering for more efficient packaging
Immunogenicity	Immune responses to capsid proteins, transgenes, or pre-existing antibodies limit efficacy	Engineering capsids resistant to neutralizing antibodies Sequential administration with low-cross-reactivity capsids Immune suppression (e.g., rapamycin, plasmapheresis) Localized administration to immune-privileged sites like the eye or brain
Vector Delivery Limitations	Risk of off-target transduction and inconsistent therapeutic efficacy	Tissue-specific promoters for higher specificity Endogenous promoter designs for sustained transgene expression Improved vector targeting through capsid modifications
Translational Hurdles	Discrepancies between animal models and human outcomes, affecting immune response predictions	Human-based studies to enhance translation accuracy Comprehensive biodistribution and shedding studies preclinically and in humans GLP-compliant data collection and analysis for preclinical safety
High Vector Doses	Large doses required for efficacy lead to toxicity and severe immune reactions	Dose optimization based on preclinical and historical clinical trial data Non-human primate studies for dosing regimens Pharmacokinetic/pharmacodynamic modeling for safer dosing
Safety Concerns	Risks include hepatotoxicity, thrombotic microangiopathy, and other adverse effects	Biomarker assessment for toxicity and disease monitoring Comprehensive preclinical safety profiling Monitoring and mitigation of genotoxicity risks

**Challenge**

**Description**

**Proposed Solutions**
